# Dynamics of collective motion across time and species

**DOI:** 10.1098/rstb.2022.0068

**Published:** 2023-04-10

**Authors:** Marina Papadopoulou, Ines Fürtbauer, Lisa R. O'Bryan, Simon Garnier, Dimitra G. Georgopoulou, Anna M. Bracken, Charlotte Christensen, Andrew J. King

**Affiliations:** ^1^ Biosciences, School of Biosciences, Geography and Physics, Faculty of Science and Engineering, Swansea University, SA2 8PP Swansea, UK; ^2^ Department of Psychological Sciences, Rice University, Houston, TX 77005, USA; ^3^ Department of Biological Sciences, New Jersey Institute of Technology, Newark, NJ 07102, USA; ^4^ Institute of Marine Biology, Biotechnology & Aquaculture, HCMR, 71500 Hersonissos, Crete, Greece; ^5^ School of Biodiversity, One Health and Veterinary Medicine, Graham Kerr Building, Glasgow G12 8QQ, UK; ^6^ Department of Evolutionary Biology and Environmental Studies, University of Zurich, 8057 Zürich, Switzerland

**Keywords:** collective animal behaviour, fish school, bird flock, goat herd, baboon troop

## Abstract

Most studies of collective animal behaviour rely on short-term observations, and comparisons of collective behaviour across different species and contexts are rare. We therefore have a limited understanding of intra- and interspecific variation in collective behaviour over time, which is crucial if we are to understand the ecological and evolutionary processes that shape collective behaviour. Here, we study the collective motion of four species: shoals of stickleback fish (*Gasterosteus aculeatus*), flocks of homing pigeons (*Columba livia*), a herd of goats (*Capra aegagrus hircus*) and a troop of chacma baboons (*Papio ursinus*). First, we describe how local patterns (inter-neighbour distances and positions), and group patterns (group shape, speed and polarization) during collective motion differ across each system. Based on these, we place data from each species within a ‘swarm space’, affording comparisons and generating predictions about the collective motion across species and contexts. We encourage researchers to add their own data to update the ‘swarm space’ for future comparative work. Second, we investigate intraspecific variation in collective motion over time and provide guidance for researchers on when observations made over different time scales can result in confident inferences regarding species collective motion.

This article is part of a discussion meeting issue ‘Collective behaviour through time’.

## 1. Introduction

The field of collective animal behaviour investigates how and why social animals coordinate their behaviour in space and time [1,2]. Research over the past few decades has shown convincingly that simple repeated interactions between individuals can produce complex adaptive patterns at the level of the group [3–6]. Individuals' sensory capabilities [7], morphology and locomotion [8] shape local interactions among individuals and the emergent properties of groups in motion, such as group density and order [3,5,9–11]. These group properties in turn affect the way information is transferred through the group and the emerging patterns of collective behaviour [9,12]. While a central tenet of collective behaviour is that individual behavioural mechanisms and the properties they produce at a group level can be similar across species and contexts [2], the specifics of interactions (that is, the behavioural ‘rules’ individuals follow) are often found to vary [10,13–18].

The similarity (or uniqueness) of local- and group-level patterns of behaviour for groups in motion across species and contexts is unclear, in part because direct comparisons of collective motion across different species or contexts are rare [19–22], and because most studies of collective motion tend to rely on short-term observations [13,23–25]. As a result, our understanding of intra- and interspecific variation in collective motion over time is limited. By quantifying how species (and populations) vary in the behavioural rules underlying collective motion, we can begin to approach collective behaviour from an evolutionary perspective [26,27]. For example, we can examine how species' ecological environments, social structures and/or phylogenetic histories shape the individual interaction rules underlying their collective motion. Despite wide interest in this topic, it is currently outside our realm of understanding, largely due to the field's current focus on the detailed study of individual populations. We must thus begin to systematically compare collective motion among different species and over different time scales [22,23,28,29].

The rarity of comparative studies on collective behaviour is surprising given the growing number of studies on collective behaviour across species, the commonality of data gathered (i.e. positional information about individuals over time) and the emphasis placed upon open data availability by researchers in collective behaviour [30,31]. Collective motion has been studied in a variety of insect (e.g. [32,33]), fish (e.g. [5,24,34]), bird (e.g. [25,35,36]) and mammal (e.g. [37–39]) species in the laboratory and the wild. In the laboratory, positional data of individuals through time is typically recorded using automated tracking of individuals from video footage (e.g. [24,34]), but inertial measurement unit (IMU) tags are also used (e.g. [40]). In the wild, studies can use video tracking (e.g. [37]) and stereophotography (e.g. [25,35]) but more commonly IMUs (e.g. [39]) and Global Positioning Systems (GPS) (e.g. [18,39,41–43]) to generate positional data. Review articles have made qualitative comparisons of collective motion across species and contexts [28], interpreting similarities or differences according to species' morphology (e.g. [44]), behaviour, social structure, communication, cognition or environment [8,28,45–47]. However, quantitative comparisons of collective behaviour across species are rare [14,19] and, to our knowledge, only a couple have focused on the specifics of collective motion [21,22].

The first goal of this paper is to begin to compare local patterns (inter-neighbour distances and positions), and group patterns (group shape, speed and polarization) during collective motion for different social species. We chose to study the collective motion of four species that are diverse in their locomotion (aquatic, aerial and terrestrial) and social structures (high fission–fusion dynamics versus stable group membership), using previously collected datasets: shoals of stickleback fish (*Gasterosteus aculeatus*) in the laboratory [24,48], free-ranging flocks of homing pigeons (*Columba livia*) [4,41], a free-ranging herd of goats (*Capra aegagrus hircus*) [39,49] and a troop of wild chacma baboons (*Papio ursinus*) [42,50]. We place data from each species within a ‘swarm space’ and suggest that this framework will be useful for generating predictions about the types of collective motion different species display. For example, researchers may have an expectation of where their data should fall in the swarm space based on the biological or ecological similarities of their study systems (for instance see [22]). Our aim is to provide a case-study with some example species; researchers can apply our swarm space framework to their own data, supporting future comparative work. When the position of new data in the swarm space does not match the initial prediction (i.e. the species or populations differ in their collective motion despite being biologically or ecologically similar), unique aspects of collective motion within and across species can be identified. Such findings may suggest future research directions, such as questions related to the response of these organisms to environmental and/or ecosystem changes.

Part of the reason that comparisons of collective motion across species are rare may be because data are collected for different time scales depending on the study species and often represent ‘snapshots’ of species behaviour [23]. For example, research undertaken in the laboratory is optimized for short periods of filming because of computationally heavy tracking [51] and benchmark studies of wild groups have used just seconds of data at particular locations [35] often restricted to short time periods or specific locations when collective motion can be observed (e.g. open areas) [25,35]. By contrast, while data on ungulate or primate group motion may extend over consecutive days or weeks (e.g. [38]) and make comparisons of behaviour in different habitats [42,52], periods of collective motion make up only a small proportion of these data.

The second goal of this paper is to investigate intraspecific variation in collective motion over time, using the four species datasets mentioned above. The stickleback fish and pigeon data come from experimentally designed observations—trials of 20 min for the fish shoals [24], and flights of just 30 s on average for the birds [41]. By contrast, the goat and baboon data come from continuous recording of their movements during the daytime—over 10 consecutive days in the case of the goats [39], and over nearly 50 days for the baboons [42]. This provides us with datasets of very different time periods across the four species, and thus different numbers and durations of consecutive periods of collective motion within these datasets. We therefore investigate if and how the metrics describing local and group patterns (group shape, speed and polarization) during collective motion change with the absolute time they are observed, or with the number of defined ‘events’ (samples) of collective movement. Our aim is to provide information about how reliable some common metrics of collective motion are when making observations over different time scales, and across different species.

## 2. Methods

### (a) Datasets

Data for three-spined stickleback fish were collected through laboratory experiments [24]. The motion of six shoals of five individuals were repeatedly tracked (using the OPENCV library in C++ on video footage with 50 fps) during free-swimming in a rectangular tank for time periods of 10 min (out of the total 20 min of each experiment) [24]. Data of homing pigeons were collected in field experiments [41], comprising GPS trajectories (sampling frequency of 0.2 s) of pigeons in flocks of 8, 10, 27 and 34 individuals being released to start their homing route [4,41]. We use ‘control’ flights from the study where pigeons were flying undisturbed [53]. The average duration of each track is 30 s. GPS data of free-ranging goats in Namibia [39] and chacma baboons in South Africa [42] were collected using SHOALgroup collars (modified F2HKv2 on the goats and F2HKv3 on the baboons). The positions of 10 adult female goats (within a herd of 16 adult females) and 13 baboons (from total of 21 adult individuals) were recorded every second for 5–6 h per day over 10 days for the goats [39] and 12 h per day over 44 days for the baboons [42].

### (b) Collective motion

**Figure 1.**
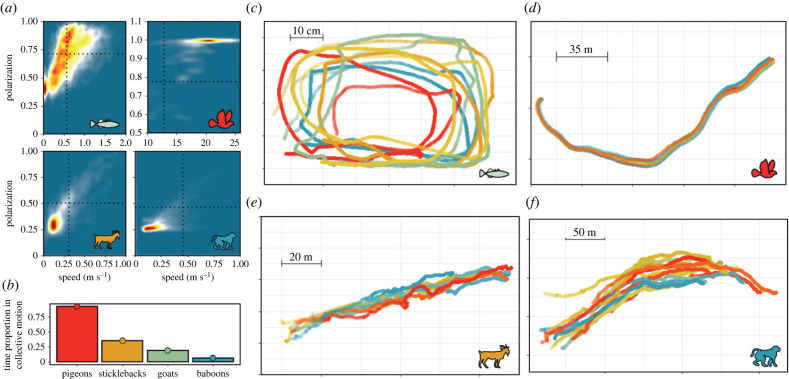
Collective polarized motion across species. (*a*) Data density over speed and polarization for each study species. The dotted lines represent the threshold we used to classify polarized collective motion in each species (pigeons = 0.05 quantiles, sticklebacks = 0.5 quantiles, goats = 0.75 quantiles, baboon = 0.9 quantiles; see also electronic supplementary material, figure S1). (*b*) Total proportion of time (from all available data) that each species spent in polarized collective motion. (*c–f*) Example tracks of a collective motion event for each species. (*c*) Tracks of 5 stickleback fish swimming in a rectangular tank for 28 s. (*d*) Tracks of 8 pigeons free flying for 31.8 s. (*e*) Tracks of 10 goats moving for 6.35 min. (*f*) Tracks of 12 chacma baboons moving for 8.65 min.

To enable among-species comparisons, we first identified periods of (uninterrupted) collective motion, defined by high speed and polarization (degree of alignment) of groups. We refer to each such period as an ‘event’ of collective motion. Thresholds for speed and polarization were selected based upon the relationship between them for each species (figure 1*a* and electronic supplementary material, figure S1). Each group's polarization was calculated as in Attanasi *et al.* [54] and the group's speed from averaging the smoothed speed of all group members. For details, see our supporting information methods. Collective motion represented different proportions of the full datasets across species, with 58 min of fish swimming over 548 discrete events, 7 min of pigeons flying over 16 events, 57 min of goat motion over 35 events and 35 h for baboon motion over 262 events (figure 1*b*). Our events are evenly distributed over time (electronic supplementary material, figure S8). Examples of collective motion events for each species are provided in figure 1*c–f*, and summary statistics for events are provided in electronic supplementary material, table S1. For sticklebacks, groups always consisted of five individuals from six different shoals, and goat data included the same 10 individuals. Pigeon flock size and composition varied, and baboon group size varied across time depending on the number of functioning collars.

Because the collective motion events we identified vary in duration (electronic supplementary material, table S1), and we aimed to compare collective motion across events (time) and species, we checked how long an event should be to be ‘representative’ of collective motion in our study groups. We therefore randomly sampled ‘snapshots’ from these events ranging from 1 to 30 s and calculated the group's polarization and shape for each snapshot separately. Group shape was defined simply, as the angle (0°–90°) between the group's heading and the direction of the short side of a minimum bounding box that includes all group members [53,55,56]. An angle of 0^°^ thus represents a group that is perfectly wider than longer, while an angle for 90° a group that is perfectly oblong (elongated in the moving direction). We normalized values by dividing angles by 90° and averaged over time to calculate the mean shape of a group during an event, which thus varies from 0 to 1. The bounding box calculations were performed using the ‘*shotGroups*’ package in R [57].

We then used a two-way intraclass correlation coefficient (ICC) (using the ‘*irr*’ package in R [58]) to test agreement between the distribution of real and sampled means for these two metrics [59]. As expected, we see an improvement of agreement (increasing ICC) with increasing duration of snapshot for all species (electronic supplementary material, figures S2 and S3). Aiming to select a single ‘minimum representative time-window’ across all four species, we based our selection on the species showing the larger increase in agreement with increasing ICC, namely pigeons (electronic supplementary material, figure S2). We thus chose a ‘minimum representative time-window’ of 15 s as the minimum sampling window needed across all four species for a sample to be representative of the real mean. According to these criteria, some events of less than 15 s (518 for sticklebacks, 2 for pigeons, 8 for goats and 88 for baboons) were not used in any further analyses. Our final dataset included approximately 15 min of collective motion for sticklebacks, 7 min for pigeons, 56 min for goats and 28 h for baboons.

Using collective motion events that are judged representative for each species (electronic supplementary material, table S1), we then calculated some local (pairwise) metrics. Specifically, we calculated the Euclidean distance between all individuals and recorded the rank distance of each neighbour from a given focal (first closest, second closest, etc). In addition to inter-individual distances, we calculated the angle between the heading of each individual and the position of each of its neighbours, namely their ‘bearing angle’, ranging from 0° (the focal individual is positioned directly behind a neighbour) to ±180° (directly in front of a neighbour to the left or to the right). We also used bearing angles to calculate ‘frontness’ which captures whether an individual is positioned in front of a neighbour, as follows:

$$f_{ij}=\frac{\left|\beta_{ij}\right|}{180^{\circ }},$$

where *β*_ij_ is the bearing angle between a focal individual *i* and its neighbour *j*. Thus, a frontness value of 1 means that the focal individual is directly in front of its neighbour (bearing angle close to 180°) and a value of 0 that the focal individual is directly behind. We calculate the average frontness of each focal individual in relation to all neighbours.

### (c) Collective motion across species

To investigate the variation in collective motion across events of our study species, we created a ‘swarm space’. This space provides a framework in which we can capture the similarities (and differences) in the characteristics of collective motion of different species. The swarm space is the result of dimensionality-reduction techniques, based on (i) local- (nearest neighbour distance (NND) and bearing angle) and (ii) group-level (polarization and group shape) measurements of each event. It is possible that, apart from these averages of each event, the temporal variation in local and group-level measurements also describe collective motion within and across species. Thus, we also calculated (iii) the temporal variation of these local and global measurements. Specifically, we estimated their standard deviation across all sampling points within an event (temporal variability in average NND, polarization and group shape). Similarly, we calculated the temporal variation in the speed of a group, and to explore individual heterogeneity in the local measurements, we calculated the standard deviation in NND and frontness within a group at each time point. From these, we calculated the average variation in frontness and NND. A large variation in frontness would reflect regular exchange of front–back positions between nearest neighbours, while large NND variation a less cohesive group (inconsistency in density). The mathematical formulae of all measurements are given in our electronic supporting information methods.

We use dimensionality-reduction methods to transform this large set of variables that describe the collective motion of a species (the 10 metrics mentioned above) into a smaller set of variables that still contains most of the relevant information in our datasets. We can thus visualize our multi-dimensional data (a dimension per metric) in fewer dimensions. Given the abundance of different dimensionality-reduction techniques, we first used perhaps the most well known in our field, the principal component analysis (PCA). We investigated the principal axes of variation in our data and created a two-dimensional ‘swarm space’. Only events longer than our minimum representative time window (15 s) were included. Metrics that did not have a significant contribution to the space were omitted from our analysis. Since the eigenvalues of each principal component created by the PCA reflect the amount of information that each component has (i.e. how much variance it captures), we focused our analysis on the ones with high eigenvalues (values less than 1 denote less variation that the individual metrics) [60]. We further labelled each principal component conceptually, depending on the metrics that contributed to it the most.

Since PCA conducts a linear dimensionality reduction aiming to retain the global structure of a dataset, it is sensitive to outliers and not as exact in capturing the data's local structure. We therefore applied another dimensionality-reduction technique to our dataset: a t-distributed stochastic neighbourhood embedding (t-SNE, using the ‘*Rtsne*’ package in R [61]). This analysis is nonlinear (and thus not sensitive to outliers), non-deterministic, and ensures that the local neighbourhoods (distance between near-by points) in the two-dimensional space are as close as possible to the one in the multi-dimensional space. We ran our t-SNE with 10000 iterations and a value of 10 for perplexity in order to focus on the local structure of our space. Overall, the two methods act complementary to each other, with the swarm space produced by t-SNE capturing the similarities between a few events, while the PCA reflects the global structure of our data.

### (d) Collective motion across time

For those individual metrics that contributed the most to the principal axes (i.e. which explained most variation in our dataset; see above), we ran a change-point and a stationarity analysis on the cumulative mean through each time series (i.e. event {1, 2, …, n}). From these analyses, we investigated if and how the cumulative mean reached a plateau when we added more events so that when there was no significant change in the mean, the given sample size provided a robust measurement of collective motion.

First, for our change-point analysis, we estimated this ‘minimum representative sample’ of events through a piecewise regression analysis (or ‘broken-line’, using the ‘*lm.br*’ package in R [62]). Specifically, we used a conditional likelihood-ratio to get a time estimate and a confidence interval (95%) within which there is a change-point between a trend and a line of slope 0 (a plateau). To ensure that the temporal order of our events did not affect our conclusions, we ran a bootstrap analysis [63] on our change-point calculation (using the ‘*boot*’ package in R [64]) with 10000 replicates. Secondly, we ran Kwiatkowski–Phillips–Schmidt–Shin (KPSS) tests (using the ‘*tseries*’ package in R [65]) in all our cumulative time series to make sure that the previously identified change-points are reflective of a substantial change in the measurement, without the time series being stationary (without a trend or seasonality over time) [66].

We thus identified the amount of time (events) needed for a confident value for each measurement for each one of our species. If no change-point is found within a time series and the time series is not stationary, this will indicate that more data are necessary for a robust estimate of this metric or that the variability in this metric is a characteristic of the collective motion in this species.

## 3. Results

### (a) Collective motion across species

**Figure 2.**
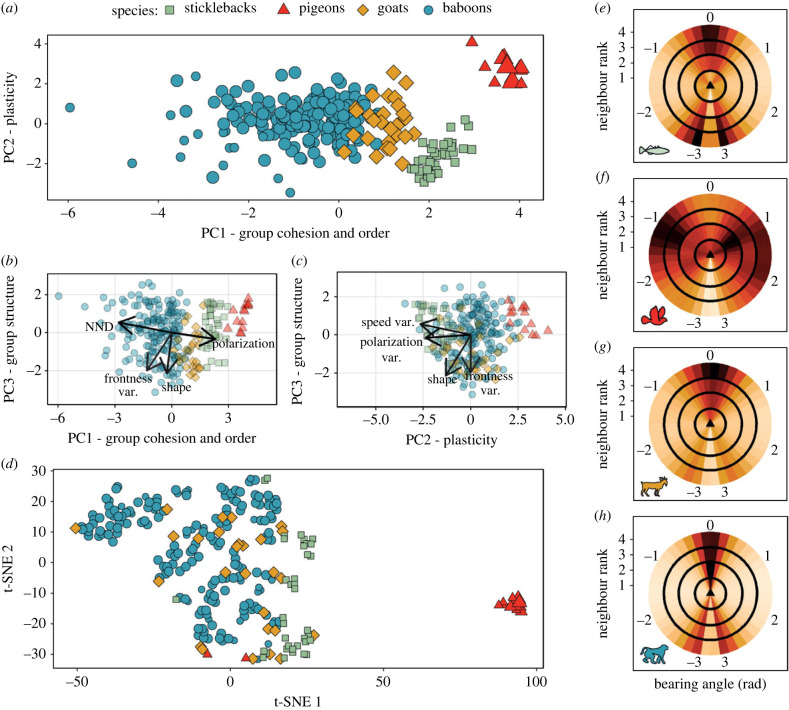
Specifics of collective motion across species. (*a–d*) Each point represents an event of collective motion (with duration longer than 15 s). The size of each point represents whether groups have less than 10 members (small), 10 to 20 (medium) and more than 20 (large). All stickleback groups consist of five individuals and all goat groups of 10. Pigeon groups vary from 7 to 33 individuals while baboon groups from 4 to 13 (with the average group size of both being 11 individuals). The group size of each point (event) is given in the electronic supplementary material, figure S6. The labels of each PC reflect the main metrics they are composed from: NND (all related metrics) and polarization (PC1), temporal variation of speed and polarization (PC2), and shape (average and temporal variation) and within-group variation in frontness (PC3). For the exact contribution of each metric in each axis, see electronic supplementary material, table S2. (*a*) Swarm space of our PCA analysis (PC1 versus PC2). (*b*) PC1 versus PC3, showing the position of all events across species according to the groups' cohesion and order and the plasticity of the groups. (*c*) PC2 versus PC3, showing the position of all events across species according to the groups’ plasticity and structure. (*d*) Swarm space of our t-SNE analysis. (*e–h*) Density maps of the position of the closest four neighbours in groups of sticklebacks (*e*), pigeons (*f*), goats (*g*) and baboons (*h*). The darker the colour of each cell the higher the density of neighbours positioned there. The polar coordinates (values −3 to 3) are the bearing angle (in rad) of neighbours with rank (topological proximity) 1 to 4 (a circle per neighbour, with the *y*-axis showing the upper end of each neighbour's circle). A bearing angle of 0 rad represents a neighbour being directly in front of the focal individual (thus here heading north).

We defined an ‘event’ of collective motion as an uninterrupted period during which individuals move (high speed) together in the same direction (polarized). We used dimensionality-reduction methods, a PCA and a t-SNE, to transform a large set of variables describing local and global characteristics of a group and create a visualization of the across and within species differences in collective motion. The first three principal components of our PCA explained approximately 70% of the variance in our collective motion event data. The eigenvalues of these first three dimensions are all higher than 1 (PC1: 3.02, PC2: 1.8, PC3: 1.48). Conceptually, this variation in collective motion reflects: the groups' cohesion and order (PC1, 33.6% variance explained; figure 2*a,b*), their temporal plasticity (PC2, 20% variance explained; figure 2*a,c*), and the groups’ structure and shape (PC3, 16.5% variance explained; figure 2*b,c*). All metrics showed an important contribution to the first three principal components, except for the average bearing angle; it explained the same variation as our measure of frontness with small contribution to the principal components (electronic supplementary material, figure S4) and was thus excluded from our analysis. The contributions of individual metrics are provided in the electronic supplementary material, table S2.

Plotting the position of each event with reference to PC1 and PC2 (figure 2*a*) and using the t-SNE (figure 2*d*) provides the ‘swarm space’. Species data are clustered together, but with some overlap, especially between the baboon and goat data (figure 2*a–c*). Group size does not seem to influence the positioning of an event in the swarm space (figure 2*a* and electronic supplementary material, figure S5), and there is little evidence of consecutive events not being independent (electronic supplementary material, figure S7). Pigeon events were the most distinct from the other species in the PCA (figure 2*a*), as well as in our t-SNE space that reflects the local similarity between events (figure 2*d*). The t-SNE analysis suggests that the goats show the largest variation in their collective motion, with their events appearing within the clusters of the other species (and even neighbouring events of pigeons). The events of stickleback fish also share similarities in collective motion not only with goats (as seen also in the PCA space) but also with some baboon events (figure 2*d*). Note that unlike the PCA space, the global structure of the t-SNE space is uninformative (i.e. the relative position of further away points is not representative of the real structure of the data) hence both analyses should be viewed together.

The variation in collective motion within and between species can be investigated further by examining the metrics that load heavily onto the principal components. For example, the nearest neighbour of an individual is more often in the forward direction for baboons, goats and sticklebacks, and in the lateral direction for pigeons (figure 2*e–h*). In fact, baboons show a strong front–back formation especially with the first two nearest neighbours (figure 2*h*). Goats also show a higher density of nearest neighbours (first–fourth) in front, but with a wider distribution than baboons (figure 2*g*). By contrast, pigeons have their nearest neighbours more often to the left or the right than in front or behind, especially for further away neighbours (fourth) (figure 2*f*). Stickleback fish show a strong difference between their first nearest neighbour (usually positioned diagonally) and the ones further away (positioned in front or behind) (figure 2*e*). These differences in how individuals are positioned is further evidenced by our new metric of frontness (see electronic supplementary material, figure S5 for the distributions of the metrics of figure 2).

### (b) Collective motion through time

**Figure 3.**
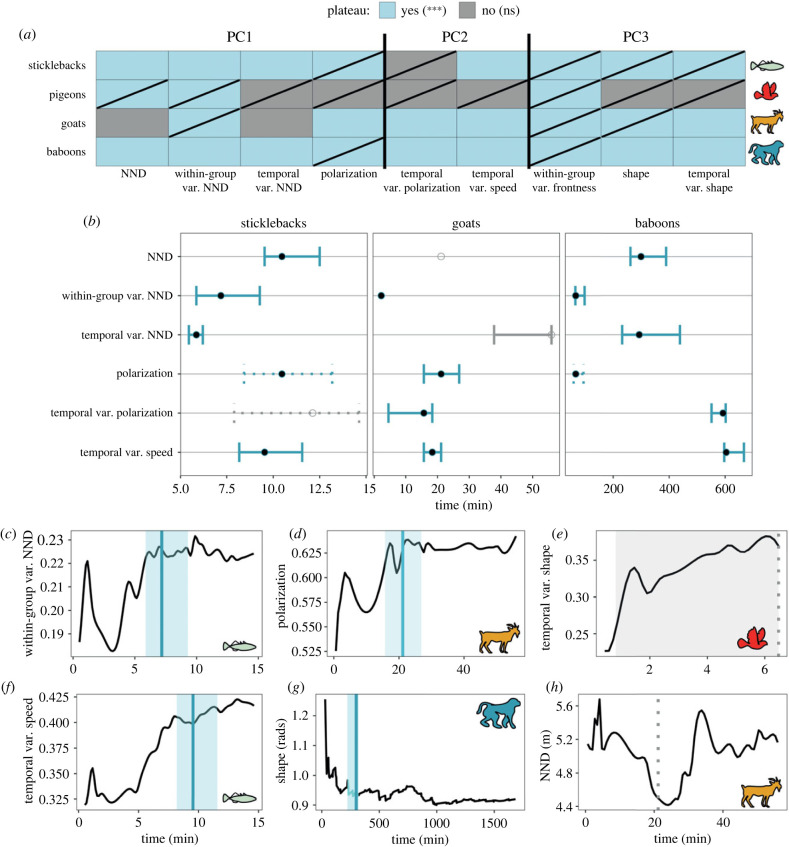
Collective motion across time. (*a*) The presence or not of a change-point in the cumulative time series of the metrics of collective motion that contribute to our swarm space (PCA). Light blue boxes show that a significant change-point to a plateau has been identified while grey boxes show that no significant change-point is found. The presence of a diagonal line in a cell denotes that this time series is stationary according to our KPSS tests (*p* > 0.05). The exact *p*-values of all KPSS tests across metrics are given in the electronic supplementary material, table S3. (*b*) Points represent the change-point estimation for when the time series stabilize. Error bars show the 95% confidence interval in estimation. Grey points and bars represent cases where the change-point was not significant (*p*-value > 0.05). Dotted error bars represent change-points in stationary time series (and, hence, are not informative). Data for pigeons are provided in the electronic supplementary material, figure S9, since all identified significant change-points are in time series identified as stationary by our KPSS analysis (*a*). (*c,d,f,g*) Examples of time series where a significant change-point (*p* < 0.05) has been identified (blue vertical line) along with the 90% confidence interval of the change-point estimation (light blue shaded area). (*e*) Example of a stationary time series (KPSS test, *p* > 0.05) in which a non-significant change-point has been identified (dotted line). (*h*) Example of a non-stationary time series (KPSS test, *p* < 0.05) in which no significant change-point has been identified.

How much data (events) are required to quantify the metrics that contribute to our PCA space? Change-points to a plateau are identified in the majority of the time series, with some however being stationary (KPSS tests with *p* > 0.05, figure 3*a*). This is particularly the case for the metrics concerning the groups' shape and structure (PC3). The estimates from our bootstrap analysis fall within the 90% confidence interval of our change-point estimation (see electronic supplementary material, figure S10).

We see a large variation in the estimated change-points across metrics and species. Change-points are identified for all metrics in the baboons, the largest dataset in our study. The fewest change-points were identified in the species with the shorter time series, i.e. pigeons. The estimated change-points in the metrics of the first two components are given in figure 3*b*. Average polarization and frontness are both stationary according to the KPSS tests, with an identified change-point in almost all species, meaning that they are perhaps the most robust metrics of collective motion even when little data is available. By contrast, temporal plasticity during events (variation in speed and polarization) required the most data to be stabilized (almost 10 h in baboons and 20 min in goats). Examples of the time series across metrics with the identified change-point and confidence interval are given in figure 3*c–h*. For the exact change-point estimation across all metrics, see electronic supplementary material, figure S9.

## 4. Discussion

Our investigation of collective motion has illustrated the similarities and differences across different time scales and for different species. There are, of course, many more and different aspects to be investigated relating to how collective motion varies across the species and time scales we study here. Nevertheless, taken together, we hope that our work can (i) guide researchers on what type of data to collect and for how long, (ii) provide insight into the self-organized dynamics that link the interaction rules that different species are likely to use and the collective patterns they exhibit and (iii) encourage future comparative work to understand the causes and consequences of collective behaviour in ecological systems. We address each aim in turn.

Our analysis placed ‘events’ (uninterrupted periods) of collective motion of stickleback fish, homing pigeons, goats and chacma baboons in a ‘swarm space’. We showed that variation in collective motion across species comes not only from absolute group characteristics (e.g. their polarization and shape) and internal structure (e.g. positioning of individuals), but also from the consistency of those characteristics across time. Based on our ICC (representative snapshots) and change-point (minimum samples size) analyses, we found that average measurements from snapshots of a few seconds are often representative of the true mean of the full time series and that most key metrics require a certain number of events (repetitions) in order to stabilize. We can thus conclude that sampling a few seconds of collective motion (in our datasets 15 s) over a larger number of days (rather than collecting long trajectories over few days), may be a more advantageous way to capture the true characteristics of a species' collective motion.

There are also important differences in the reliability of metrics over time, which may be affected by differences in the number of events identified for each species within our datasets. For example, the baboon data took the most time to stabilize, but this time is still a small proportion of the whole time series available (figure 3*g*). Therefore, a smaller dataset (less events) than the one we used here should provide accurate results for baboons, if similar metrics are investigated. Stickleback fish and goat data also tended to stabilize over time but required a much higher proportion of our total data available. Therefore, in the case of the goats and fish, it seems that the datasets available (time, events) were sufficient for estimating the metrics we presented. In contrast with the other species, the pigeon time series tended to be stationary over our increasing sample size (that is, from the first event studied). This indicates that the collective motion of the pigeons showed less variation than our other species, but whether this was because of the context the data were collected in (initiating their homing flight) or the small duration of the time series remains to be tested.

Based on our findings, we can also hypothesize on the underlying interaction rules of group members, which were not considered explicitly here. For instance, the specifics of locomotion may be responsible for many differences we see across species [9], especially for pigeon flocks that showed the largest differentiation from the other three species. The wide shape of pigeon flocks with nearest neighbours positioned on the side of a focal individual may emerge from a cohesion mechanism based on acceleration [18] in combination with turning to avoid collisions [4]. Stickleback fish, goats and baboons share a more oblong group shape and front–back internal structure (figure 3*e,g,h*), but the nearest neighbour of sticklebacks is rarely directly in front of a focal individual (in contrast with the goats and baboons). This may again be attributed to turning to avoid collisions while swimming, while goats and baboons may mediate collision avoidance mostly by changes in their speed. Additionally, having a nearest neighbour positioned directly in front may indicate pairwise attraction dynamics instead of attraction to the centre of a number of neighbours (as identified in some fish and bird species [13,67]). Given that differences in interaction rules across species and contexts are increasingly found in the literature [10,13,16,68], keeping a species- and context-specific level seems necessary when drawing conclusions about collective behaviour. For instance, whether the behaviour of sticklebacks is representative of fish species (see for instance [21]) or whether the behaviour of pigeons while homing is representative of pigeons during free flight are assumptions that need more data to be tested.

Adding more data from events of collective motion will now enable across-species comparisons and deepen our understanding of self-organized patterns of collective behaviour. Just considering our small sample of four species, we can begin to hypothesize about ‘higher level’ collective behaviour, such as collective navigation, decision-making and escape. For instance, if we take the metric of ‘frontness’, which we introduced here, its variation could be related to the function of collective motion in different species or be an inevitable outcome of their environment [24,42,52,69]. If differences are functional, we could speculate that species with a clear front–back structure in motion may have stronger ‘leadership’ dynamics [24,42,70,71] associated with collective motion that is linked to decision-making concerning navigation. By contrast, species with low frontness could be organized to maximize information transfer [54] with collective motion linked to anti-predatory functions [41,43,53]. If differences are the result of environment, then front–back structures may reflect constraints of moving through largely two-dimensional terrestrial environments that present more physical and energetic constraints upon movement in space [52,72] compared to three-dimensional environments that are more open [73]. Furthermore, our t-SNE space that highlights the similarities between events of different species also suggests potential links in dynamics of collective motion across species. For example, the surprisingly wide distribution of events of collective motion of goats in the t-SNE space (i.e. their resemblance with events of all other species in our datasets) indicates the variation in collective motion that goats exhibit, making them an interesting, and to date not well studied, species.

We now encourage colleagues in collective behaviour research to use our swarm space framework with events of collective motion of their study species. This will allow us to identify ‘model’ species of collective behaviour that are representative of their taxa, but also those species that are functionally unique. A wide range of important ecological questions can then be addressed in terms of the diversity of collective behaviour (where events and species fall in the swarm space). Simple questions around the variation in collective motion for studies in controlled laboratory environments versus experiments or observations conducted in the wild can be tackled. But perhaps most interesting are questions about the evolutionary and ecological determinants of diversity in collective behaviour and the potential links to ecosystem level processes [23,29,74–77]. It will be possible to begin to test whether specific collective motion is correlated to locomotion (e.g. swimming versus flying), or socio-ecological variables (e.g. social system), and measure how strongly phylogenetic relatedness predicts the distribution of collective behaviour across species.

There are challenges associated with future comparative work, though, as highlighted by our study. Differences in the specifics of each species' dataset may pose constrains for the generalization of conclusions reached from their comparison. First, the stickleback data are collected in the laboratory [24] while pigeon, goat and baboon data are collected in the field [39,41,42]. Second, the duration of our pigeon trajectories is much shorter than the trajectories of the other species (this however may not affect our across-species analysis, since pigeons show the highest percentage of collective motion, figure 1*b*). Third, the sampling frequency used across our datasets varies. Given that the frequency of observations at each study tends to be made at biologically relevant scales, sampling frequency may not have a significant effect on how groups are placed within the swarm space, but it should nevertheless be considered in future research. Fourth, in the datasets of sticklebacks and pigeons, each group consists of a different combination of individuals, while in goats and baboons the same individuals are being tracked as a group. Thus, we may underestimate the variability in collective motion for goats and baboons. Lastly, as is common when deploying tags in wild group-living animals, the data of baboons and goats do not cover all the individuals of their groups. It therefore remains to be seen how these differences may introduce noise or confound future comparative work. However, if we continue to focus on characteristics of collective motion (local and global metrics), these potential issues can be overcome, either by directly testing for their effects in any analyses (e.g. laboratory versus wild data; see above), or keeping these issues in the forefront when making interpretations.

Overall, our study provides a starting ground for future research on collective behaviour under a more unifying framework. Identifying the differences in self-organized dynamics across species is a necessary step before generalizing identified patterns from one or a few species. Similarly, identifying the time scales of each characteristic of collective motion can facilitate our search for the interaction rules that underlie group-level patterns and can help ensure the validity of past findings. Knowledge of dynamics of collective motion across species and time, as presented in our work, can inform the design of data collection in future research (in a manner that relates to research in other species) which in turn can ensure valid comparisons across study systems.

## Data Availability

Data on metrics of collective motion across species and time are stored in the Zenodo repository: https://doi.org/10.5281/zenodo.7457770, and the code to reproduce our figures and analysis is available at the GitHub repository: https://github.com/marinapapa/ColMotion-Species-Time (https://doi.org/10.5281/zenodo.7566410).
